# Case Report: Lomustine overdose in a 15-year-old, healthy adolescent—a prescription failure

**DOI:** 10.3389/fped.2024.1339597

**Published:** 2024-02-12

**Authors:** Ole Lindner, Natalia Rotari, Ayami Yoshimi, Charlotte M. Niemeyer, Simone Hettmer

**Affiliations:** ^1^ Division of General Pediatric and Adolescent Medicine, Department of Pediatrics and Adolescent Medicine, Faculty of Medicine, University of Freiburg, Freiburg, Germany; ^2^ Division of Pediatric Hematology and Oncology Medical Center, Department of Pediatrics and Adolescent Medicine, Faculty of Medicine, University of Freiburg, Freiburg, Germany

**Keywords:** lomustine, chemotherapeutic, overdose, pediatric, oncology, adverse events, prescription

## Abstract

Lomustine is an oral chemotherapy drug commonly used in pediatric neuro-oncology. We report on a 15-year-old formerly healthy boy, who was erroneously prescribed lomustine instead of an antibiotic for tonsillitis. He subsequently suffered from prolonged bone marrow aplasia with secondary fever in neutropenia and ubiquitous bleeding. Bone marrow regeneration started approximately 7 weeks after lomustine intake. No other permanent organ damage has been detected thus far. Oral chemotherapeutic drugs should only be prescribed by experts and dispensed in the smallest possible pack size.

## 1 Background

Lomustine is an oral chemotherapy drug, which is typically used as part of combination therapies for brain tumours or brain metastases (1). It is a well-established alkylating agent and usually administered as a single dose of 80–110 mg/m^2^ every 6 weeks. The drug is given orally, often in an outpatient setting. Due to the expected bone marrow toxicity and the risk of severe nausea and/or other serious side effects, close monitoring of the patient's condition by an experienced oncologist is recommended. In fact, prolonged and/ or deep thrombocytopenia may result in delays in therapy and/ or bleeding complications.

## 2 Case

**Figure 1:**
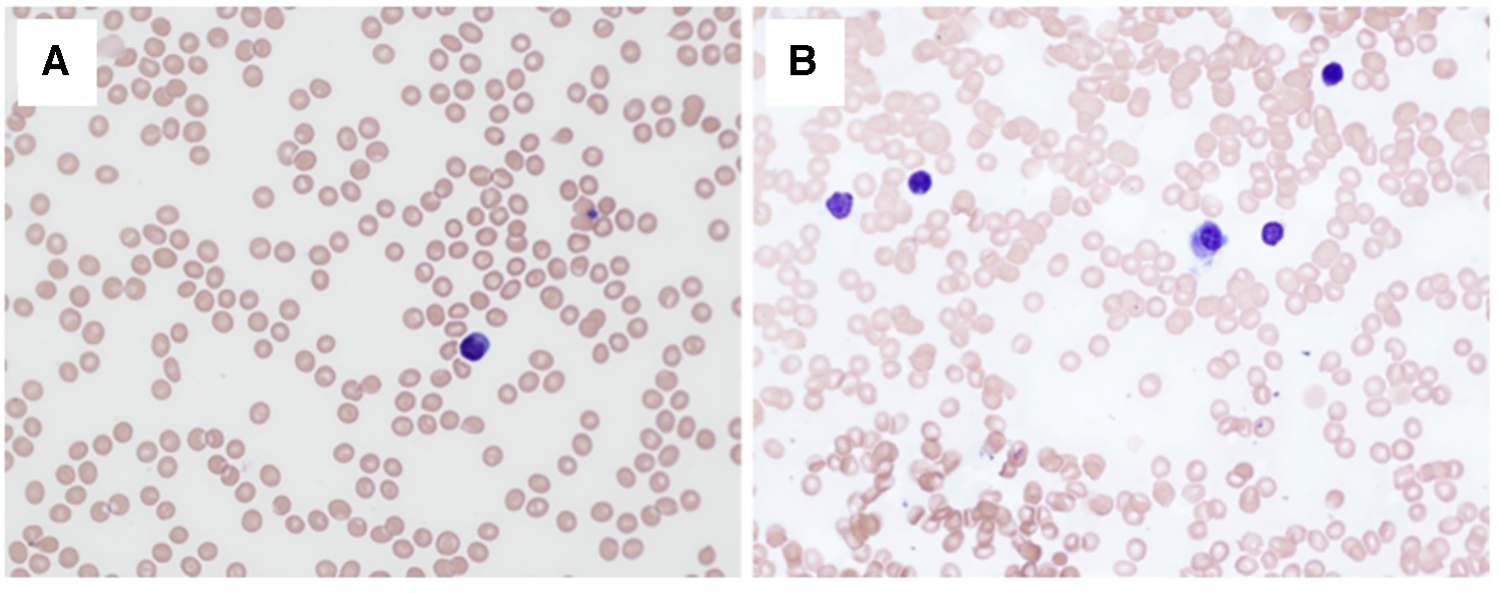
Blood and bone marrow cytology. Peripheral blood smear (**A**) and bone marrow aspirate smear (**B**) on day 26 after first dose of lomustine showing tricytopenia and bone marrow aplasia (May-Grunwald-Giemsa stain, ×40 objective, the total magnification ×400).

**Figure 2:**
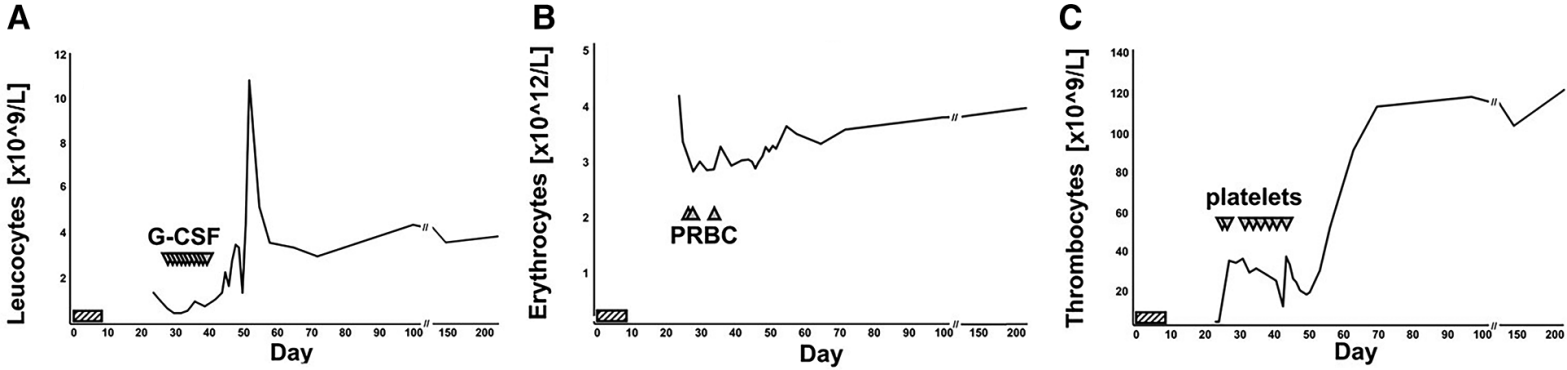
Numerical hematology parameters over time. Leukocyte (**A**), erythrocyte (**B**) and thrombocyte (**C**) counts displayed over time. Lomustine intake on days 1 to 7 was marked by striped horizontal bars. G-CSF infusions (**A**), packed red blood cell (PRBC) transfusions (**B**) and erythrocyte transfusion (**C**) was marked by triangles. The patient was admitted to hospital on day 24 and discharged on day 52. The most recent set of blood counts was obtained on day 219 post lomustine intake.

A 15-year-old formerly healthy boy presented to his general practitioner because of a sore throat and fever. Streptococcal A tonsillopharyngitis was diagnosed. Due to suspected allergy to penicillin, the provider intended to prescribe cefaclor (Cec®) as an alternative antibiotic. However, a prescription for lomustine (Cecenu®) was issued erroneously, with instructions to take it three times a day. The physician reported later, that she accidentally selected the drug in the alphabetical list of drugs provided by the electronic prescriping tool. In addition, the pharmacist failed to perform the required plausibility check and did not inform the patient's guardians on the type of medication/dosing recommendations when dispensing it. Over the next 7 days the patient took 20 capsules, equaling 800 mg (445 mg/m^2^) lomustine in total. This represented a cumulative dosage more than four times higher than the regular single dosage (80–110 mg/m^2^). On day 13 after the first lomustine intake, the patient was seen by the general practitioner again with persistent fever (maximum temperature 40.2 °C), extreme fatigue and cough. He tested positive for influenza A and received supportive treatment at home. On day 22 after the first lomustine intake, the patient developed ubiquitous petechial bleeding, dizziness, repeated episodes of fainting and painful blood blisters in his mouth. On day 24 after the first lomustine intake, he was admitted to our inpatient service. By that time, his labs revealed severe thrombocytopenia (2 × 10^9^/L), leucopenia (1.40 × 10^9^/L), neutropenia (0.46 × 10^9^/L) and anemia (erythrocytes 4.05 × 10^12^/L, Hb 12.1 g/dl). He complained of increasing abdominal pain. Bone marrow aspirates and trephine biopsies were consistent with severe aplasia (Figure 1). Upon further questioning of the patient and his parents, the erroneous intake of lomustine became apparent. Due to the relatively short half-life of lomustine (48–72 h) and the delayed diagnosis (20 days after the last intake of lomustine), no attempt was made to determine the plasma concentration of lomustine. No activated charcoal was administered. Over the course of the following month, the patient was given granulocyte-colony-stimulating factor (G-CSF, 263 µg/dose), and he required transfusion of 10 platelet concentrates and 6 red cell concentrates. The platelet nadir was observed on day 24 and the leukocyte/neutrophil nadir on day 31 after the first dose of lomustine (Figure 2). The patient developed lobar pneumonia without need for respiratory support. He was treated with broad-spectrum antibiotics (piperacillin-tazobactam, tobramycin and, subsequently, meropenem) and amphotericin. On day 49 after the first lomustine dose (hospital day 25), his neutrophil count reached 1.93 × 10^9^/L under ongoing exposure to G-CSF. G-CSF was stopped one week later. Six months after lomustine intake, the patient was symptom-free, but his blood counts had not recovered fully with persistent thrombocytopenia, leukopenia and macrocytosis (erythrocytes 3.85 × 10^12^/L, Hb 12.8 g/dl, MCV 97.4 fL, MCH 33.2 pg, thrombocytes 123 × 10^9^/L, leucocytes 3.80 × 10^9^/L, neutrophil count 1.71 × 10^9^/L). 12 months after lomustine intake, blood counts normalized, while macrocytosis was still present (thrombocytes 190 × 10^9^/L, erythrocytes 4.75 × 10^12^/L, Hb 15.5 g/dl, MCV 96.6 fL, MCH 32.6 pg, leucocytes 6.57 × 10^9^/L, neutrophil count 3.29 × 10^9^/L). Lung, liver and renal function tests were within normal range, and no other sequelae were found. He continues to receive regular screening for late effects in our outpatient clinic. The parents took legal action against the medical provider on behalf of their son. A report was submitted to the national pharmacovigilance authorities. The case was discussed in our hospital's morbidity and mortality rounds. The prescriber and the pharmacist, who dispensed the drug, were involved in the discussion. Informed consent for publication was obtained from the patient and his parents.

## 3 Discussion

To our best knowledge, this is the first reported case of a lomustine overdose in a previously healthy individual. The 15-year-old previously healthy adolescent took 445 mg/m^2^ lomustine resulting in prolonged bone marrow aplasia, grade IV mucositis, pneumonia and ubiquitous bleeding. A literature search was performed (using the keywords lomustine, overdose) and revealed seven other published cases of lomustine overdosing; all affecting patients with advanced cancer (Table 1) (2–7). All but one of them suffered from brain tumors and received lomustine as part of a palliative chemotherapy regimen (2, 4–6). One published case was due to another serious prescription error by a general practitioner, who prescribed lomustine on a daily schedule (5). All patients with lomustine intoxication experienced prolonged myelosuppression and severe thrombocytopenia. Higher lomustine doses appeared to correlate with earlier onset and longer duration of aplasia (2–6), presumably raising the risk of lethal outcome (4). There are no reports of long-term adverse effects following lomustine overdose. We cannot predict the likelihood of late sequelae and note persistent macrocytosis 12 months post lomustine intoxication with concern.

**Table 1:** Patients with lomustine overdose reported in the literature^a^.

Reference	Age (years)/gender	Malignant diagnosis	Lomustine cumulative dose (exposure time)	Bone marrow nadir^b^	Outcome
Foon and Haskell (2)	59/m	Colorectal carcinoma metastatic to liver and lung	550 mg/m (7 days)	d20–d35	Alive at 22 months post lomustine overdose
Hörnsten et al. (3)	62/m	Hodgkin's disease	300 mg/m^2^ (15 days)	d30–d45	Alive at 4 years post lomustine overdose
Trent et al. (4)	28/f	Anaplastic astrozytoma	825 mg/m^2^ (7 days)	d14–d50	d29 liver failure, d44 enzephalopathy, d50 respiratory failure, d59 death
Abele et al. (5)	35/f	Anaplastic astrozytoma	805 mg/m^2^ (7 days)	d10–d45	Alive at 11 months post lomustine overdose
Büyükçelik et al. (6)	38/m	Glioblastoma	400 mg/m^2^ (4 days)	d16–d35	Death at 9 months post lomustine overdose due to disease progression
Büyükçelik et al. (6)	48/m	Glioblastoma	330 mg/m^2^ (4 days)	d14–d42	Alive at 30 months post lomustine intake
Wirsching et al. (7)	71/m	Glioblastoma	350 mg/m^2^ (4 days)	d10–d37	Dead at 4 months post lomustine intake due disease progression
Our case	15/m	None	445 mg/m^2^ (7 days)	d21–d49	Alive, persistent mild bone marrow suppression at 6 months post lomustine intake

f, female; m, male; d, days after first lomustine intake.

^a^ Adapted from Wirsching et al. (7) and Abele et al. (5).

^b^ Myelosuppression: Platelets <100 × 10^9^/L, leukocytes <3.0 × 10^9^/L, absolute neutrophil count <1.5 × 10^9^/L or hemoglobin <9.0 g/dl.

Serious (medication) errors, such as the one described here, are typically caused by a series of faulty actions, also known as the Swiss cheese model. If there is an unfavorable combination of multiple causal factors, individual errors result in damage, accidents or catastrophic events. Orally administered chemotherapeutic drugs are generally well-received, as they are easy to take at home and allow higher quality of life than intravenously administered anti-cancer drugs. However, there is a substantial risk of accidental overdosing. Additional safety measures could be established easily and reduce potential risks. Firstly, the packaging size of lomustine may be reduced to include a single dose. All providers—including support staff and pharmacists—should be required to actively monitor the plausibility of any prescription. Automatic safety checks could be incorporated in the electronic prescription tool. Last but not least, errors in medical procedures should be discussed openly and published whenever possible to allow for improvements in standard procedures.

## Data Availability

The raw data supporting the conclusions of this article will be made available by the authors, without undue reservation.
